# Diet supporting therapy for inflammatory bowel diseases

**DOI:** 10.1007/s00394-021-02489-0

**Published:** 2021-03-31

**Authors:** Justyna Kikut, Nina Konecka, Maciej Ziętek, Danuta Kulpa, Małgorzata Szczuko

**Affiliations:** 1 Department of Human Nutrition and Metabolomics, Pomeranian Medical University, Szczecin, Poland; 2 Department of Applied Neurocognitivistic, Pomeranian Medical University, Szczecin, Poland; 3 Department of Perinatology, Obstetrics and Gynecology, Pomeranian Medical University, Szczecin, Poland; 4 Department of Genetics, Plant Breeding and Biotechnology, West Pomeranian University of Technology, Szczecin, Poland

**Keywords:** IBD, CD, UC, Inflammation, Diet ingredients

## Abstract

**Purpose:**

Non-specific inflammatory bowel diseases (IBD) include Crohn's disease and ulcerative colitis. More and more often attention is paid to the possibility of dietary support for inflammatory bowel diseases.

**Methods:**

The following review article considers the role of dietary components in the treatment of IBD as: pteridines, probiotics, bovine immunoglobulin, vitamin D, omega-3, flavonoids, polyphenols, curcumin and phosphatidylcholine. The article also discusses plant raw materials of arjuna, soy protein and nettles, trying to summarize their effect on quenching the inflammatory process within the intestines. This review focuses on the possibilities of dietary components and supplementation use to improve the pharmacotherapy response as well as the general clinical patients’ condition.

**Results:**

The mechanism of action of supportive therapy is based on reduction in oxidative stress, maintaining the adequate balance between Th1 and Th2 lymphocytes by affecting cytokines, increasing riboflavin supply for macrophages, increasing expression of vitamin D receptor, regulation by decreasing the expression of NF-κB in liver cells and ability to inhibit the COX2 entrance and inactivate prostaglandins that are involved in the inflammatory process and 12-lipoxygenase pathway inhibition.

**Conclusion:**

Considering clinical researches, it seems that the use of the above-mentioned ingredients in the diet of patients suffering IBD may positively influence the treatment process and maintenance of remission.

## Introduction

Non-specific enteritis is associated with persistent digestive system inflammation [1]. For that reason, the most important IBD pharmacological treatment goal, is to reduce inflammatory reaction. Currently, both anti-inflammatory and immunosuppressive therapies are on use [1]. Children less than 10 years of age are increasingly diagnosed with IBD. In addition, IBD appearance in childhood is linked to a more severe course of illness [2]. Unfortunately, the disease affects many aspects of a young people's life—education, nutrition, mental state and normal growing. The IBD pathogenesis is still not well understood [2]. Both the environment, intestinal barrier status as well as genetics and immune response have an impact on the disease development [3].

Microbes that cross the intestinal barrier, activate the immune system by releasing a cascade of anti- and proinflammatory signals. Leukocytes migrate across the intestinal epithelial barrier, causing the organism’s balance disruption and the T cells immune response is excessive [3]. Interleukin 12 activates Th1 cells, which in turn produce large amounts of interferon gamma. In contrast, the release of IL-4, IL-5, IL-13 is attributed to Th2 cells activities. In patients suffering from Crohn's disease, the increased IL-2 and IFN-γ levels have been also observed. However, in *Colitis Ulcerosa*, NK (natural killers) cells release excessive amounts of Th2 cells associated cytokine IL-23. Hence the conclusion, that Crohn's disease is associated with an immune response mediated by Th1 cells, whereas UC—by Th2 cells [4]. A significant relationship has been also found between the diet and IBD. Results from the review article demonstrated a higher risk of IBD exacerbation with higher red meat consumption as well as processed food protein and alcohol. A total fat content in the diet and omega-3 to omega-6 ratio, have been also found to be associated with a significantly increased risk of IBD relapse [5]. The nutrition and the use of anti-inflammatory ingredients such as turmeric, omega-3 or vitamin D have also a positive effect on suppression the inflammatory process, associated with IBD [5]. A constantly increasing inflammatory bowel disease prevalence, increasingly occurring in younger group of individuals, leads to supposition that an adequate modified diet implementation may support the pharmacological treatment, improving patients’ clinical condition [5].

## Current therapy methods of inflammatory bowel diseases

Current treatment of inflammatory bowel diseases (IBD) is based on the use of corticosteroids, aminosalicylates, thiopurine, immunomodulatory and biological drugs [6].

The longest-term therapy is based on sulfalazine and 5-aminosalicylic acid (masalazine) use that have a positive effect on inflammatory process quenching. Glucocorticosteroids are mostly administered in active phases of disease, due to their high efficacy in remission induction. Additionally, they rapidly relieve symptoms of disease and are relatively safe, in condition to be given in recommended doses and in short period of time as well. Immunosuppressive therapy is usually reserved for severe stages of disease and often implemented in patients with corticosteroid resistance. Thiopurines, to which belong azathioprine and 6-mercaptopurine, are mainly used as a maintenance therapy. Biological therapy is based on antibodies activity, obtained in genetic recombination process, thereby affecting by pro-inflammatory mediators blocking, especially TNF-α [7].

Despite the many therapeutic options, drugs do not always give the effective result. Therefore, increasingly a special attention is paid to the positive impact of nutrition, especially to benefits of so-called nutraceutical products consumption in the IBD patients’ group, when taking consideration for inflammatory process [8]. Also, the role of nutrition becomes an important part of therapeutic process. It has been shown that too high intake of omega-6 acids relative to omega-3 is associated with an increased level of inflammatory marker—C-reactive protein (CRP) [9]. A normal fatty acid ratio of omega-6 to omega-3 intake in diet is 1: 4–5 [10]. Nutrients can modulate inflammation by affecting cytokine secretion or uptake of certain side products [9]. In addition, numerous studies indicate positive effects of the diet that have an impact on reducing inflammatory process. A study conducted by Akbaralay et al. showed that a long-term healthy diet, in accordance with the principles of rational nutrition, reduce the IL-6 levels, which has a significant impact on the course of inflammatory reaction [11]. In turn, poor dietary habits and inadequate nutrient intakes, leading to post meal hyperglycemia is considered to be one of the main causes of systemic inflammation [12].

## Pteridines in the fight against inflammation

Recent studies demonstrate, that pteridines analogs occuring in a natural way, affect the immune mechanisms that regulate the TNF-α levels [13]. In vitro studies proved that synthetic pteridine analog 4AZA2096 administration inhibits strongly the lipoplisaccharide induced TNF-α production, thus suppressing the inflammation reaction [13]. The transcription nuclear factor κB (NF-κB) is a nuclear factor that binds to the promoter of the immunoglobulin gene in B lymphocytes [14]. Therefore, it plays an important role in not only immune but also inflammatory processes. In addition, it shows an activity in cell proliferation and apoptosis processes [14]. It was found that in inflammatory bowel diseases the NF-κB activity degree is correlated with inflammation severity [15]. Increased level of NF-κB expression in macrophages of mucous membrane is accompanied by increased secretion of TNF-α, IL-1 and IL-6 [15]. Additionally, macrophages and monocytes are sensitive to vitamin B2 deficiency, under which conditions, they lose the pathogen phagocytosis activity [16]. Furthermore, reactive oxygen species (ROS) stimulate the nuclear factor NF-κB, which in turn stimulates the genes encoding TNF-α and IL-1β expression, leading finally to increase these pro-inflammatory cytokines concentrations [17]. Tumor necrosis factor seems to play an important role in the CD pathogenesis. The mechanism of TNF-α action is wide, it inter alia induces the adhesion factors expression that contribute to the development of cell inflammation through the macrophages activation and release of other pro-inflammatory cytokines, e.g., interferon-γ [18]. Further in vitro studies indicate the anti-inflammatory effect of pteridines. The synthetic pteridine 4AZA2096 administration to the macrophages has demonstrated an inhibition of proinflammatory cytokines secretion such as TNF-α and IL-6 [19]. Both the congenital and adaptive immune response stimulate Th1 cells activation leading to release large amounts of cytokines, e.g. IL-2, IL-12, IL-18, IFN-γ. IFN-γ, as a pro-inflammatory cytokine, induces an anti-neoplastic and antimicrobial defense, interalia by stimulating neopterin synthesis, as a consequence of guanosine-5′-triphosphate (GTP) induction in macrophages. Macrophages and other ROS cells interfere with the NF-kB activation signal, resulting in proinflammatory cytokines production, among others TNF-α. However excessive accumulation of ROS leads to increased Th1 immune response [20]. Husain et al. has also demonstrated that neopterin is secreted throughout activated interferon by both T lymphocytes and macrophages [21]. What's more, macrophages can also secrete neopterin by stimulating homocysteine. Husein et al. conducted a study in 70 patients with Crohn's disease, 52 patients with UC, and 144-person control group. The aim of the study was to determine a new marker of diseases’ activity–neopterin concentration. It was found that neopterin concentrations in faeces increased in people with active and inactive CD but were not statistically significant, while in patients with UC, the serum neopterin concentrations were significantly higher in UC active phase when compared to those in remission. Therefore, neopterin presence in stool may indicate remission or exacerbation in UC but is unuseful as a marker in CD. It is probable, that the degree of neopterin concentrations increase in stool may be related to the location and severity of disease in the colon [21]. Maier et al. have analyzed in the study food preservatives activities such as curcumin, sodium benzoate and propionic acid [22]. These compounds have been shown to influence the neopterin synthesis by inhibiting its production via IFN-γ [22]. It is also believed that neopterin is a reliable marker in the evaluation of Th-1 type immune activation. Its measurement in body fluids is proposed to be a marker of disease progression [23].

## Probiotics

According to the FAO/WHO, probiotics are considered as live microorganisms that have a beneficial effect to the host organism, when given in appropriate doses. The probiotic bacteria group include mainly lactic acid bacteria and some non-pathogenic fungi, e.g., *Saccharomyces cerevisiae*. Among different probiotics properties, in addition to their beneficial effect on intestinal microflora, the ability to modulate cytokine production is indicated. The joint supply of yeast *Saccharomyces boulardii* and mesalazine reduced the rate of relapse in 6.25% of subjects compared to the placebo group, in which the recurrence rate reached 37.5% [24]. However, another probiotic yeast *Saccharomyces boulardii* research indicate that the probiotic administration alone has no positive effect on patients suffering CD. Therefore, studies results cannot be considered to be consistent [25]. In a study performed on intestinal epithelial human cells, a preparation containing three strains of bacteria: *Lactobacillus rhamnosus lr32, Bifidobacterium lactis bl04* and *Bifidobacterium longum bl05* has been used (strains present in dietary supplement of l-theanine, l-cystine, vitamin b2 with lactic acid bacteria. This preparation caused a significant increase in the anti-inflammatory cytokine IL-10 concentration and significant decrease in the concentration of pro-inflammatory cytokines IL-1β and IL-6, leading to inflammatory process reduction [26]. In a Fedorak’s et al. study a multi-vaccine VSL # 3 containing *Lactobacillus* (*pracesei* DSM 24733, *plantarum* DSM 24730, *acidphilus* DSM24735, *delbrueckii* subsp. *bulgaricus* DSM 24734), *Bifidobacterium* (*longum* DSM 24736, *breve* DSM 24731) and *Streptococcus salivarius* (subsp, *thermophilus* DSM 24731) strains has been used. In a group of 120 Crohn's disease patients, that have been included in a randomized trial, a recurrence incidence reduction in endoscopic imaging after previous surgery was performed. After the preparation administration (1 sachet 2 times a day), the second look colonoscopy was performed on day 90th and 1 year after surgery. There were no significant differences between the after preparations’ administration group and the placebo group (3 g of corn starch). There was a lower frequency of relapses in the group, where a VSL # 3 was administrated for 1 year, which fact may encourage further research to conclude a causal association between preparation administration and course of disease [27].

The *Lactobacillus plantarum* belonging to Firmicutes group has a beneficial health effect in inflammatory bowel diseases as well. The strain provides a balance between Th1 and Th2 lymphocytes’ concentration, by affecting cytokines such as TNFα, IL-1β, IL-6, IL-10, IL-12, IFN-γ. In addition, it blocks the cyclooxygenase 2 pathway in Th1. It also modulates the immune response of lymphoid and epithelial cells in the gut, which has been proved both in vitro and in vivo studies [28]. In the active phase of inflammatory bowel diseases, an elevated macrophages and T lymphocytes level is observed. They are activated by excessive production of IL-1β, TNF-α and ROS [18]. In the next examination, a colon tissue sample was taken in adults with an active form of UC. Both in macrophages and T cells an increase in IL-10 production and subsequently inflammatory-quenching reaction was observed, which indicates a positive effect of the *Lactobacillus plantarum* strain [29]. The limitation of this study was the small number of patients from whom the biological samples were collected.

*Lactobacillus rhamnosus* (LGG) and *Lactobacillus plantarum* strains have also been demonstrated to increase the vitamin D-VDR receptor expression. Probiotics reduced also an intestinal inflammatory reaction by increasing the number of Paneth cells that participate in intestinal defense reactions [30]. In another study, the administration of *Lactobacillus plantarum K8 lysate* reduced the proinflammatory secretion of cytokines IL-6 and TNF-α [31]. In contrast, Yin et al. have proved that the anti-inflammatory micro integral membrane protein (MIMP) isolated from *Lactobacillus plantarum* reduces the proinflammatory cytokines such as IFN-γ, IL-17 or IL-23 levels, which contribute to the IBD induction, while increasing the concentration of anti-inflammatory cytokines, to which the IL-4 and IL-10 belong [32].

In studies performed on mice, it has been demonstrated, that *Lactobacillus plantarum CRL2130* administration, being a strain overproducing riboflavin, increase significantly the IL-10 levels in both serum and intestinal tissue cells, thereby reducing the inflammatory process. In addition, when compared to the control group (mice that did not receive a strain), the diarrhea incidences’ reduction, an intestinal mucosa condition improvement and an intestinal villi protective effect were noticed [33]. The results seem promising, but Derwa et al. indicates that there is no advantage over the use of probiotics compared to placebo in inducing remission in active UC. However, VSL3 # has demonstrated the benefit of a probiotic over placebo in inducing UC remission. Additionally, probiotics may be as effective as 5-ASA in preventing exacerbations in UC. When inducing remission in the active phase of CD, there were no benefits of using probiotics compared to placebo [34]. Kruis et al. conducted study on a group of 327 UC patients (double-blind trial) in which the first group of patients (*n* = 162) received probiotics at a dose of 200 mg/day and 2 groups (*n* = 165) received 500 mg mesalazine 3 times/day. The aim of the study was to confirm evaluate the effectiveness of 2 methods in the prevention of recurrence of the disease. The study lasted 12 months during which clinical, endoscopic and histopathological activity of the disease was assessed. The probiotic group showed relapse in 36.4 vs. 33.9% in the mesalazine group. *E. coli* Nissle 1917 shows confirmed efficacy and safety in maintaining UC remission comparable to the effects of mesalazine treatment [35].

The 2017 recommendations of the World Gastroenetrology Organization regarding the use of probiotics in IBD patients indicate that in case of Crohn's disease there is no evidence that the supply of probiotics has a beneficial effect on maintaining remission. In ulcerative colitis, these recommendations indicate that some probiotics are safe and effective in achieving better response to treatment and inducing disease remission [36].

Undeniably, many studies show the benefits of different probiotic strains in IBD patients. It is important to note, however, that clinical trials differ in dose, composition and group of individuals or material that included the studies. The strain should also be adapted not only to the disease but also to its phase.

## Bovine immunoglobulin

Serum-derived bovine immunoglobulin (SBI) is a preparation, which belongs to the special-purpose medical food. Supplementation with SBI 5 g/day for 12 weeks maintains healthy intestinal immune system by binding a broad range of microbes and toxins within the gut lumen in patient group with CD and UC [37]. The probable mechanism of SBI’s action is based on immunoglobulin connection with antigen. A so created connection, being a large complex, cannot pass through epithelium. In addition, the formed complexes do not allow antigens to encounter dendritic cells, even when the epithelium is damaged [38].

A study conducted on 50 patients diagnosed with IBD, showed a clinical condition improvement in 49% of subjects, just after SBI administration for one week. After 12 weeks’ SBI treatment, the total of patients being in better condition rose to 76% [37]. In the next study, in case of a patient resistant for UC standard therapy, a clinical condition improvement has been observed just after SBI administration for 2 months [38]. The applied therapy concerned the supply of 5 g SBI 4 times a day for 7 days and then for 5 g a day, which prevented the recurrence of disease in this period. Another study showed a reduced daily stools’ number in IBD patients treated with bovine immunoglobulin preparation. This led to clinical conditions’ improvement, as well as better patient’s quality of life [39]. In another study, conducted on 40 patients diagnosed with IBD, resistant to pharmacological therapy, the intensity and incidence of gastrointestinal symptoms were analyzed. During the 6-week investigation, patients were given orally 5 g of SBI daily. In relation to the pre-treatment assessment with SBI, a significant reduction in the occurrence of nausea and diarrhea was observed [40]. In case described by Soriano et al. a single case for UC teens after 5 g SBI per day during 2 months, a decrease in the PUCAI rate, the subsided of poles and the absence of blood in the faeces was observed [41]. Interestingly, Shafran et al.' study showed a possible reduction in the cost of standard treatment in Patients with IBD receiving SBI. The subjects received 5 g SBI per day for 8 weeks, which resulted in health improvement and reduced the cost of waiting for their biological therapy [42].

In conclusion, although there are currently few studies on the impact of SBI in IBD patients and the study groups do not cover a large number of patients, they seem promising. They show a reduction in gastrological symptoms among patients and an improvement in their quality of life. It is important to add that all the studies presented above were conducted with the participation of people. It is worth to consider the introduction of SBI as a nutritional aid in IBD therapy.

## Vitamin D

Vitamin D has a protective effect on the intestinal mucosa, among others by increasing the proteins’ expression, responsible for tight connections creation. Vitamin D affects also an epithelial integrity in a process of inhibiting intestine epithelial apoptosis, by which influences intestinal inflammation and healing process. Vitamin D increases the suppressive mediators of inflammation production such as IL-4, IL-5 and IL-10, but reduces IL-12, IFN-γ, IL-2, IL-17, TNF-α concentrations that are responsible for stimulating inflammation [43].

Analyzing tissues samples in colonic biopsies in patients with IBD, a decreased concentration of vitamin D receptor (VDR) was showed, while the level of pro-inflammatory cytokines such as TNF-α and IL-1β was observed to be high, which led to conclusion that the VDR can be displaced by cytokines in immune-mediated diseases. The mechanism of vitamin D activity includes decreasing the proinflammatory cytokines, e.g., IL-1, IL-6, IL-8 and IFN-γ and TNF-α, while enhancing protective immune responses [44]. 1,25 D3 has also an ability to inhibit COX2 and inactivate prostaglandins involved in inflammation process [45]. In a study conducted on 34 persons, of whom the 19 had vitamin D deficiency, the incidence of subjects with active phase of UC disease has been observed to be higher in the vitamin D deficiency group. Also the Mayo Score for ulcerative colitis activity in the group with vitamin D deficiency was to be higher when compared to normal vitamin D levels’ group as well as the need of having steroid therapy in group of humans with vitamin D deficiency was statistically higher. Interestingly, the study did not show a statistically significant relationship due to the season change [46]. In Lairds’ et al. study, conducted on 957 older adults aged of 60 years and more, the metabolite of 25 (OH) vitamin D was measured by means of liquid mass spectrophotometry and the level of cytokines IL-6, IL-10, TNF-α and CRP, has been analyzed. Patients treated with glucocorticosteroids, cytokine modulators and suffering from dementia were excluded. The obtained results showed a negative significant correlation between the vitamin D deficiency degree and the IL-6 and CRP concentrations, the IL-6 to IL-10 ratio was disturbed as well. These data confirm the role of the vitamin D axis in mucosal barrier development, integrity and healing capacity and indicates the need to normalize the vitamin D level to ensure the proper functioning of the immune system [47]. The vitamin D influence on the course of CD disease was also evaluated by Jorgensen et al. in a randomized study. For a period of 12 months, 46 CD patients received an oral formulation containing 1200 IU of vitamin D3, while 48 patients received a placebo. The study showed a lower rate of relapse in patients treated with vitamin D3, compared with placebo group. However, the rate of relapse decreased from only 29–13% [48].

Metaanalysis of Del Pinto et al. indicates that vitamin D as a therapeutic agent has proven to be promising in reducing the frequency of relapses and improving the quality of life in IBD. In turn, strong evidence has been found indicating that a group of patients with IBD have twice the chance of developing vitamin D deficiencies compared to healthy control [49].

In conclusion, vitamin D has a documented effect on the immune system. In the case of inflammatory bowel diseases, its deficiency may influence the intensity of inflammation. Normalization of its level may help in correct function of the immune system and healing of the intestinal mucosa. ESPEN recommendations for vitamin D indicate the need to monitor patients in the active phase of the disease and treated with corticosteroids for vitamin D 25(OH) levels and supplementation should be used [50].

## Omega-3 acids

Polyunsaturated fatty acids (PUFA) from the omega-3 family, have a confirmed immunomodulatory and anti-inflammatory effect. Omega-3 fatty acids compete with omega-6 acids in the cyclooxygenase and lipoxygenase pathways for leukotrienes and prostaglandins synthesis. These acids may also reduce the hydrogen peroxide production by reacting with Reactive Oxygen Species (ROS). Hydrogen peroxide is one of the Nuclear Factors’ κβ activator [51]. Experimental animal studies that received varied PUFA content diets within 4 weeks showed changes in the concentration of different inflammatory markers.

A diet with omega-3 acids predominance has reduced the COX-2 (cycloxygenase) expression in colon and decreased the IL-6 and TNF-α production as well. A diet with omega-9 acids predominance has been also found to decrease the colon IL-6 production [52]. The next experimental studies performed on rats, took consideration for omega-3acids supply followed by 5-ASA (5-aminosalic acid) therapy, where subjects have been administered with drug alone or omega-3 acid alone, as controls. The results indicate the omega-3 and 5-ASA combination to be more beneficial in therapy, due to more effective inflammatory response inhibition by reducing the NF-κB activation. In addition, the omega-3 and 5-ASA are more effective together at reducing the NF-κB activation when compared to higher 5-ASA dose, administered alone. It seems that the implementation of omega-3 supplementation may allow to reduce the dose of used medicaments [53]. A systematic review on the effectiveness of n-3 acid use in CD remission (*n* = 1039) and UC (*n* = 138) showed a lack of sufficient data to commission the use of omega-3 to maintain remission of both diseases [54]. ESPEN recommendations do not recommend the inclusion of n-3 supplementation to maintain remission in patients with IBD [50]. In conclusion, n-3 acids play an important role in reducing inflammation, however, the strongest effect is observed in the therapy combining pharmacological treatment and n-3 supplementation.

## Nutraceuticals

Nutraceuticals according to Dr. Stephen L. DeFelice’s definition are defined as a food or part of a food, such as a dietary supplement, that has a medical or health benefit, including the prevention and treatment of disease [55]. These include isolated food constituents, herbal products, especially phytochemicals and dietary supplements. Nutraceuticals contain biologically active substances that can affect physiological and metabolic functions and have a beneficial effect on the human organism [55].

**Eupatilin** and quercetin-3-β-d-glucuronopyranoside belong to flavonoids.

In the Joo et al. study, the eupatilin extract is an ethanol extract made from the dried parts of the plant *Artemisia vulgaris* L. In this investigation eupatillin was administered orally to rats with induced acute colitis for 48 h. After this period, an inflammatory response was analyzed by myeloperoxidase (MPO) level measuring, nitric oxide production, TNF-α expression and total glutathione concentration. The results demonstrated that MPO level, nitric oxide production, TNF-α expression decreased in a dose-dependent manner, opposite to glutathione level that increased. In addition, the given eupatilin extracts and quercetin-3-β-d-glucuropyranoside reduced oxidative stress and improved inflammatory response [56]. In vitro studies conducted by Zhou et al. have demonstrated that eupatilin inhibits the NF-κB activation both in intestinal epithelial cells, in macrophages and in experimental models, where colitis have been induced. In addition, this compound influences the NF-κB suppression in activated macrophages and reduces the oxygen free radicals’ production. Improvement of intestinal barrier function was also observed through the hermetically connections sealing in tight junctions cells [57].

In summary, studies carried out on animals and in vitro cultures show the positive effect of eupatilin extract on inflammation reduction. However, further human studies are needed to confirm the validity of its use in IBD patients.

**Apigenin** is another compound belonging to flavonoids, which occurs naturally in artichokes, cheese or Mexican cultivar of oregano [58]. In Mascaraque et al. research, the rats with induced colitis received orally apigenin K (its soluble form). The inflammatory response was examined with use of microscope and biochemical analyze. In the microscopic imaging, the inflammation area was observed to be reduced, and in biochemical parameters, the myeloperoxidase, TNF-α and IL-6 concentrations decreased. Thus, the anti-inflammatory effect of apigenin in inflammatory bowel diseases in an animal model have been demonstrated [59]. Other studies conducted also on rats with experimental colitis evaluated the apigenin and *Dracocephalum kotschyi* Boiss hydroalcoholic extract supply and prednisolone as the standard drug for comparison. The biochemical evaluation of the colon inflammation was carried out by measuring the myeloperoxidase (MPO) activity. This research has demonstrated after 5 days treatment, an anti-inflammatory potential of apigenin in experimentally induced colitis, and its beneficial effect was comparable with that of prednisolone [60]. Ben-Arye et al. conducted a randomized double-blind placebo-controlled trial, using wheatgrass juice—*Triticum aestivum*. Twenty-three patients with diagnosed UC were randomly assigned to the wheatgrass juice group or to the placebo group, respectively. The study lasted for a month and patients received daily doses of juice or placebo. After this time, the disease activity index was calculated. The results showed that the juice intake was associated with a significant reduction in the rate of disease activity and reduction in severity of rectal bleeding [61].

The use of apigenin may have a positive effect on the reduction of disease activity in patients with UC. In addition, it may reduce rectal bleeding in this group. However, there is a lack of data confirming apigenin activity in patients with CD.

**Polyphenols** contained in the grape extract, mainly anthocyanins, flavonol glycosides and hydroxycinnamic acids have a protective effect on ulcerative colitis in mice. A study conducted by Boussenna et al. has shown that the 21-day supply of a polyphenol-rich preparation to UC-induced mice, reduces the proinflammatory cytokines production and influences the MPO and antioxidant enzymes activities [62]. Polyphenols derived from apples have also a positive effect on inflammatory process. This potential has been confirmed in D'Argenio et al. research, who administered rectally apple methanol extract to rats with experimental colitis for 14 days. The results have shown a reduction of inflammatory process activity as well as normalization of inflammatory markers values, such as IL-1β, IL-1α, IFN-γ, IL-6, TNF-α [63]. Different polyphenols can also affect the human intestinal microflora. A study with caffeic acid, chlorogenic acid, catechin, epicatechin, rutin, naringenin, daidzein and quercetin, which were stored in dimethyl sulfoxide (DMSO) in temperature—20 °C was carried out on Caco-2 cell line enterocytes. The influence of polyphenols on the intestinal bacterial growth and their adhesion to enterocytes was investigated. The influence of polyphenols on the growth of *Lactobacillus rhamnosus, Escherichia coli, Staphylococcus aureus* and *Salomnella typhimurium* was observed. The results showed an effect of all tested polyphenols, excluding rutin, on the viability of intestinal bacteria. Naringerin had the most significant inhibitory effect on the four tested bacteria, while all polyphenols influenced on Staphylococcus aureus inhibition. On the other hand, rutin enhanced the *Lactobacillus rhamnosus* adhesion to enterocytes [64].

Studies with the purple potato extract, rich in polyphenols, showed an improvement in the condition of intestinal epithelium. The intestinal barrier function was improved by the activation of AMP-activated Protein Kinase (AMPK). Activated AMPK in turn mediated an increase in Caudal type homebox 2 (CDX2) expression, which in turn is a transcriptional factor responsible for intestinal epithelial cells differentiation [65]. AMPK is an energy metabolism regulator having an important role in maintaining metabolic homeostasis [66, 67]. It influences the epithelial barrier by differentiating, forming and maintaining it. Additionally, AMPK strengthens its action by increasing CDX2 expression [65, 67].

**Curcumin** belongs to the curcuminoid family and is a natural compound that can be found, among others, in the turmeric root. Suskind et al. conducted studies on pediatric patients being in IBD remission or having mild disease symptoms. All of patients received curcumin as a standard pharmacotherapy supplementation. Supplementation lasted a total of 9 weeks, during which the preparation was administrated in variable doses. The results show that curcumin has been well tolerated in all patients, and the PDCAI (Pediatric Crohn's Disease Activity Index) and PUCAI (Pediatric Ulcerative Colitis Activity Index) was improved. These indicators consider the biochemical blood parameters (hematocrit, albumin), abdominal pain occurrence, the daily stools quantities, general well-being and parenteral symptoms [68]. Another placebo-controlled study has been conducted in adults with UC where curcumin was administrated for disease remission maintaining. The supplementation lasted 6 months, administrated in oral dose of 2 g daily. The calculated CAI (Clinical Activity Index) has shown significantly lower values in preparation receiving group, compared to the placebo. The studies’ conclusions are consistent and confirm that curcumin use in patients with IBD may be a safe and effective pharmacotherapy support, by dint of tumeron activity [69]. A meta-analysis of the use of curcumin and mesalazine was carried out by researchers from the USA. While the literature analysis using the MEDLINE, Pubmed and Embase databases searching in December 2017, investigators compared studies with curcumin and mesalazine use versus placebo. Randomized trials in 142 patients have shown that the use of a two-component treatment is associated with a higher incidence of disease remission. In addition, basing on endoscopic examination, the disease remission and patients’ condition improvement has been observed to occur more frequently in curcumin treated group, compared to placebo [70]. In another study, 89 patients with UC participated in a randomized, double-blind placebo-controlled study. 45 patients received supplementation with curcumin at a dose of 1 g a day in combination with mesalazine or sulfalazine, a 44 person placebo group received the drug alone. The study lasted a total of 6 months, the clinical activity index (CAI) and the endoscopic index (EI) were calculated at the patients’ admission, then every 2 months and at the end of the study. The results showed a significant difference between curcumin and placebo. The combination of pharmacotherapy with curcumin reduced relapses in this group of patients [71].

The use of curcumin as an anti-inflammatory agent is common, and curcumin is widely used in therapy of many diseases. Jimenez-Flores et al. conducted a study in diabetic mice, supplemented with curcumin for a period of 8 weeks. The investigators concluded that curcumin regulates by decreasing the expression of NF-κB in the liver of mice [72].

Curcumin has a strong anti-inflammatory effect. Its supplementation combined with pharmacotherapy in IBD therapy has very positive effects—it increases the percentage of remissions obtained and reduces the frequency of exacerbations.

## Phosphatidylcholine

Phosphatidylcholine (PC) is the main lipid of the gastrointestinal mucosa layer constituting a barrier against bacterial invasion, thus providing an anti-inflammatory effect. Studies conducted in patients with UC have shown their low mucus PC content. Similar results were obtained by German researchers who observed that patients with UC have significantly lower content of mucus PC, when compared to patients with CD and healthy control group [73, 74]. However, it is estimated that in persons with Crohn's disease, the amount of PC is reduced by up to 70%, which may additionally contribute to the inflammation occurrence [74]. In addition, clinical trials made on the Caco-2 cell line showed that using the slow-release PC form supply has a beneficial therapeutic effect on colonic mucus [75]. Amadei et al. also suggest that PC administration to defected mucus layer in IBD patients may be a promising treatment strategy [76]. A randomized, double-blinded placebo-controlled trial performed by Stremmel et al. was carried out on 60 patients with UC. Patients were randomly divided into two groups: receiving a slowly released phosphatidylcholine (PC) supplement and a placebo group. After 3 months observation, 58 patients underwent a colonoscopy (2 patients have refused). The results showed induction of clinical remission in 53% of PC group patients while remission was achieved by 10% in the placebo group [77].

In summary, it is the missing component of the intestinal mucosa in patients with inflammatory bowel disease. Therefore, a therapy to supplement it, administered as a supplement, may be promising. However, further research is needed on a larger group of patients.

***Terminalia arjuna***, commonly known as Arjuna, is a plant which root and fruit are widely used in medicine. It is a large deciduous tree, belonging to the *Combretaceae* family, widespread in India, Burma, Mauritius and Sri Lanka [78, 79]. It has antiseptic, immunomodulating and antioxidant properties. The Arjuna’s root contains as much as 60–70% of polyphenols, especially those having an anti-cancer activity of flavonoids, tannins and triterpenoids [80]. It also includes such amino acids as tyrosine, histidine, cysteine and tryptophan [78]. It is recommended as a supporting treatment in inflammation processes, ulcers and diarrhea. The Cota et al. study aimed to investigate the anti-inflammatory activity of *T. arjuna* in rats with experimentally induced colitis. The animals were administered *Arjuna* in various doses (500, 250, 125 mg/kg) for 28 days, after which the extent of intestinal tissue damage was evaluated by macroscopic and histological assessment, as well as concentrations of inflammatory mediators, e.g., TNF-α, IL-1β, Il-6. The results indicate a reduction in the macroscopic and histological characteristics of intestine inflammatory process. In addition, the myeloperoxidase (MPO) and nitric oxide (NO) concentrations decreased. *Arjuna* has been shown to have a protective effect, leading to glutathione, superoxide dismutase and catalase concentrations decrease. Analysis of gene expression of proinflammatory mediators—TNF-α, IL-1β, Il-6 showed a decrease in the expression of these genes, which had a beneficial effect on intestinal microflora. The best anti-inflammatory effects were obtained at a dose of 500 mg/kg of the preparation [79].

Animal studies show the potential effect of *T. arjuna* on the reduction of inflammation. However, further research is needed to confirm its effectiveness in humans.

## Soy protein

Investigations suggest a potential soy protein anti-inflammatory effect. Metzger et al. researchers from the US, curried out the analysis, whether the use of a diet enriched with soy protein in an animal model with chronic colitis can affect the reduction of inflammatory process. The animals were given a 35% soy protein content diet for 28 days. Histological examinations of both the intestinal and bone tissues have shown their structures improvement related to dietary supply. In addition, the TNF-α concentrations were reduced. For resolving question what the mechanism is by which white soy influences TNF-α, further research is needed. It seems that a soy protein rich diet can strongly affect the environment of both the stomach and intestines by modulating the lymphatic structure [81].

## Common nettle

*Urtica dioica* belongs to the popular annual plants group. It is used in medicine mainly due to its ability to increase the binding capacity of iron and vitamins B9 and B12 in blood [82]. It contains flavonoids, phenolic acids, silicic acids, scopoletin having anti-cancer activity and reduced diuretic effect. It is also characterized by a high concentration of rare elements such as titanium and silicon [83].

In a study carried out by Nematgorgani et al. in 64 persons, dried hydroethanolic nettle leaf extract administered in the form of tablets was used for a period of 3 months. 30 patients received nettle preparation containing 400 mg of nettle extract, 3 times a day, while the 29 patients received placebo. The high-sensitivity CRP serum levels as well as platelet count demonstrated a significant reduction. However, there have been observed a superoxide dismutase (SOD) increase, being responsible for free radicals’ elimination. In addition, in patients with coexisting inflammatory bowel diseases, a clinical health improvement was noted. The quality of patients’ life measured using the disease dedicated IBDQ-9 questionnaire has also improved significantly [84].

In ex vivo human platelets investigations, it was shown that nettle root extract inhibited thromboxane production as well as the release of pro-inflammatory LPS induced chemokines and COX2 expression in intestinal epithelial cells. The herbal extract has also inhibited the 12 LOX pathway. *Urtica dioica* influences an increase of intestinal epithelial cells chemokine secretion, which not only stimulates a signal transduction but also supports the maintenance of epithelial integrity and intestinal mucosal defensive ability. Therefore, the nettle plant modulates the immune system response not only the sick patients, but also healthy individuals in extract concentration of 200 mg/L [82].

In connection with the above information, it seems important to include mentioned nutraceuticals in patients' diet. Especially since they can be consumed with appropriately selected products in the daily diet. Nutraceuticals administered in the form of supplements or directly with nutrition can support pharmacological treatment, support the maintenance of correct integration of the intestinal epithelium and have an effect on the reduction of inflammation. However, further research is needed to determinate the mechanism of action of diet compounds in CD and UC.

**Fig. 1:**
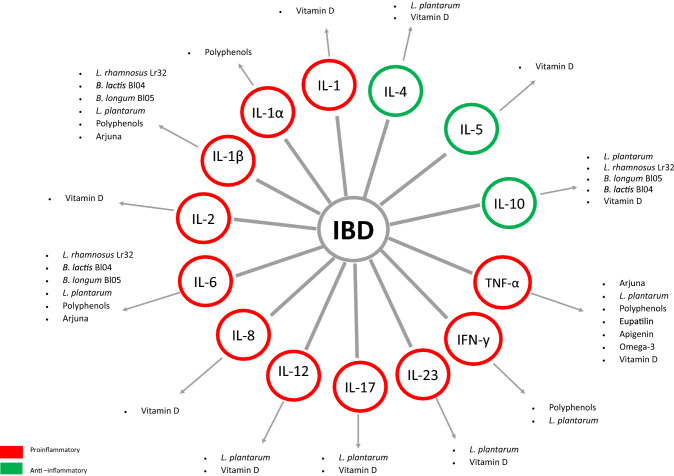
Diet supporting therapy for inflammatory bowel diseases (IBD)—the influence of the substances described on pro-inflammatory (red color) and anti-inflammatory (green color) mediators is presented. *TNF-α tumor necrosis factor α; *IFN-y* interferon gamma; *IL* interleukin

In summary, there is a wide range of diet supporting therapy for inflammatory bowel diseases that is worth considering in the active phase of disease Fig. 1.

In conclusion, the studies analysis of dietary interventions in CD and UC available in the medical literature databases has shown a positive diet effect on clinical patient’s condition in both diseases. Nevertheless, further research is needed to determinate the mechanism of action of diet compounds in CD and UC. An adequate dietary intake in patients, rich in components that have been discussed in the recent article, undoubtedly supports the pharmacological treatment of IBD. The analysis of collected studies is presented in Table 1.

**Table 1:** Summary of articles investigating the effect of probiotics on inflammation, n-3 acids, vitamin D and nutraceuticals on inflammation

Author	Study type	Number of patients	Time	Intervention	Efficacy outcome(s)
Probiotics					
Fedorak et al. [27]	CD patients	*n* = 120 (*n* = 59 vs. placebo *n* = 60)	1 year	VSL#3 (1 sachet 2 times a day)	Lower frequency of relapses in the study group
Pathmakanthan et al. [29]	Cells from normal and active mucosa from colon adults with UC	–	–	*Lactobacillus plantarum* 299	Macrophages and T cells an increased in IL-10 production and subsequently inflammatory-quenching reaction
Wu et al. [30]	Mice Human intestinal epithelial cells	*n* = 12 (*n* = 6 with VDR and *n* = 6 without VDR)	24 days	*Lactobacillus rhamnosus* (LGG) and *Lactobacillus plantarum* (LP)	Increased VDR expression Increased the number of Paneth cells-reduced inflammation
Ahn et al. [31]	Rats	*n* = 47 (control group *n* = 10; colitis control group *n* = 9; group with live *Lp. plantarum* K8 1 × 10^9^ CFU *n* = 9; group with *Lb. plantarum* in 1 × 0^9^ CFU *n* = 10; group with *Lb. plantarum* in 1 × 10^10^ CFU *n* = 9	14 days	*Lactobacillus plantarum* K8	Reduced IL-6 and TNF-α in the colon Reduced colon shortening, edema, mucosal damage High-dose of lysate increased colonic expression of receptor-2 mRNA
Yin et al. [32]	Mice	*n* = 18 (control group *n* = 6; DSS-treated group (*n* = 6); MIMP-treated group (i = 6)	14 days	*Lactobacillus plantarum*	Reduced proinflammatory cytokines IFN-γ, IL-17 or IL-23 Increased IL-4, IL-10 Improved gut microbiota dysbiosis
Levit et al. [33]	Mice	*n* = 25 (5 groups of 5 animals	5 days	*Lactobacillus plantarum* CRL2130 vs control group	Increased the IL-10 levels Diarrhea incidences’ reduction An intestinal mucosa condition improvement An intestinal villi protective effect
Derwa et al. [34]	Systematic review and meta‐analysis	–	–	–	No advantages over the recommendations for the treatment of placebo in the induction of remission in active UC VSL3 # has demonstrated the benefit of a probiotic over placebo in induced UC remission Probiotics may be as effective as 5-ASA in preventing exacerbations in UC Inducing remission in the active phase of CD: there were no benefits of using probiotics compared to placebo
Kruis et al. [35]	Patients with UC	*n* = 327 (study group *n* = 162; group with only drugs *n* = 165)	12 months	Study group: 200 mg/day probiotics Second group: 500 mg meslazayny 3xday	*E. coli* Nissle 1917 wykazuje potwierdzoną skuteczność i bezpieczeństwo w utrzymaniu remisji UC porównywalnie do wpływu leczenia mesalazyną
Bovine immunoglobulin					
Shafran et al. [37]	Patients with IBD	*n* = 45 (CD group *n* = 38; UC group *n* = 7)	12 weeks	5 g SBI/day	Clinical condition improvement
Beauerle et al. [38]	Single case study—patient with UC	*n* = 1	2 months	First week—5 g SBI × 4/day After first week 5 g SBI/day	Clinical condition improvementin case of a patient resistant for UC standard therapy Improved Mayo UC score
Shaw et al. [39]	Survey with IBS/IBD patients	*n* = 595 (*n* = 344 IBS; n = 251 = IBD)	–	SBI	Reduced daily stools’ number Improvement quality of life
Liaquat et al. [40]	Patients with IBD (who were refractory to standard treatment)	*n* = 40	6 weeks	5 g SBI/day	Reduction nausea, diarrhea
Soriano et al. [41]	Single case study—pediatric patient with UC	n = 1	2 months	5 g SBI/day	Blood in the stools, and diarrhea resolved PUCAI score decreased
Shafran et al. [42]	Patients with IBD	*n* = 21 (CD *n* = 14; UC *n* = 4)	2 months	5 g SBI/day	Possible reductions in the cost of standard care have been demonstrated in IBD patients receiving SBI
Vitamin D					
Blanck et al. [46]	Patients with UC	*n* = 34	–	–	Higher Mayo Score for ulcerative colitis activity in the group with vitamin D deficiency Vitamin D deficiency is associated with a greater need for steroid treatment
Jorgensen et al. [48]	Patients with Crohn disease in remission	*n* = 94 (46 CD patients/48 CD patients-placebo)	12 months	1200 IU vitamin D3/day	Oral supplementation increased serum vitamin D levels Relapse rates in patients supplementing with vitamin D3 decreased from 29 to 13%
Del Pinto et al. [49]	Meta-analysis	–	–	–	Vitamin D as a therapeutic agent has been shown to be promising in reducing the frequency of relapses and improving the quality of life in IBD IBD patients have been shown to have a twofold higher risk of developing vitamin D deficiencies compared to control
Omega-3 acids					
Charpentier et al. [52]	Rats with induced colitis	*n* = 50 (divided into 5 groups: TNBS, n-3, n-6, n-9, control)	4 weeks	Diets with several fatty acid proportions: n-3, n-6, n-9	In diet with OMEGA3-reduce iNOS, COX-2 expression in colon; decrease IL-6, TNF-α production In diet with OMEGA9-decrease the colon IL-6 No differences between groups in levels: claudin-1 and occludin
Mbodji et al. [53]	Rat with induced colitis	–	14 days	Omega3 acid (fish oil-rich formula) or isocaloric and isolipidic oil formula + 5-ASA	Combination of omega 3 with 5-ASA treatment—reduction of NF-κB activation (together they are more effective than a higher dose of the drug alone)
Turner et al. [54]	Systematic review with IBD patients	CD *n* = 1039; UC *n* = 138	–	–	Insufficient data to order the use of omega-3 to maintain remission of both diseases
Eupatilin					
Joo et al. [56]	Rat with induced colitis	*N* = 6 w 8 grupach	48 h	Artemisia vulgaris L EIE—ekstrakt z Artemisia asiatica (100 mg/kg) lub EIQ—ekstrakt z Rumex aquaticus (30 mg/kg) 5-AS- kwas 5-aminosalicylowy (25 mg/kg)	Reduced MPO activity, nitric oxide production, TNF-α expression in a dose-dependent manner
Zhou et al. [57]	Mice with induced colitis	5 groups *n* = 10 animals per group	7 days	Eupatilin (10 or 20 mg/kg/day)	Inhibits NF-κB activation in both intestinal epithelial cells, in macrophages, and in experimental models where colitis has been induced
Apigenin					
Mascaraque et al. [59]	Rat with induced colitis	6 groups *n* = 4–6 per group	All treatments started 2 d before colitis induction	apigenin K(1, 3 or 10 mg/kg)	Reduced inflammation area Decreased: myeloperoxidase, TNF-α and IL-6
Sadraei et al. [60]	Rats with experimental colitis (9 group *n* = 6 in each group)	3 grupy dostały rosnące dawki Kotschyi; 3 kolejne apigeninę; kolejne 3 kontrola	5 days	Apigenin (5, 10, and 20 mg/kg)or Dracocephalum kotschyi Boiss (10, 20, and 40 mg/kg) hydroalcoholic extract—ere administered orally 2 h prior to induction of colitis	Lower score values of macroscopic and microscopic characters Decreased MPO Reduced the total colitis index Best effect was achived with dose of 5 mg apigenin
Ben-Arye et al. [61]	Patients with active UC	*n* = 23	1 month	100 cm^3^ wheatgrass juice—*Triticum aestivum* or placebo	Reduction in the rate of disease activity Reduction in severity of rectal bleeding
Polyphenols					
Boussena et al. [62]	UC-induced rats	*n* = 40 (divided into 5 groups)	21 days	Polyphenol-rich red grape pomace extracts (GPEs)	Reduces the proinflammatory cytokines production and influences the MPO and antioxidant enzymes activities
D'Argenio et al. [63]	Rats with TNBS-induced colitis	*n* = 16 (divided in 2 groups, *n* = 8 in each group)	14 days	Apple methanol extract (1 mL/die of APE 10−4 M)	reduction of inflammatory process activity as well as normalization of inflammatory markers values, such as IL-1β, IL-1α, IFN-γ, IL-6, TNF-α
Parkar et al. [64]	Caco-2 enterocytes	–	–	Hydroxycinniaminc acids; flavonoids; glycosides; flavanone; isoflavones; flavonol 10,30, 100 µg/mL After 1 h of incubation of *Lactobacillus rhamnosus, Escherichia coli, Staphylococcus aureus* and *Salomnella typhimurium* was added to cells	All polyphenols inhibited *Staphylococcus aureus* adhesion The most sensitive to polyphenols is *S. aureus* High antibacterial activity: flavanone, flavanol Low antibacterial activity: flavonols, isoflavones, glycosides
Curcumin					
Suskind et al. [68]	Children with IBD	*n* = 11 (UC *n* = 5, CD *n* = 6)	9 weeks	500 mg capsules (for all patients, no placebo) 2 razy dziennie przez 3 tygodnie 1 g dwa razy dziennie od 3 tygodnia 2 g dwa razy dziennie od 6 tygodnia	PDCAI, PUCAI was improved,
Garg et al. [69]	Patients with UC—review	*n* = 89 (treatment n = 45 placebo *n* = 44)	6 months	2 g curcumin/day (oral dose)	Lower CAI index compare to placebo group
Iqbal et al. [70]	Patients with UC—review	*N* = 142 (study group *n* = 71; *n* = 71 placebo)	–	Curcumin + mesalazine	Higher incidence of disease remission versus placebo Endoscopic remission
Hanai et al. [71]	Patients with UC	*n* = 89 (*n* = 45 study group; *n* = 44 placebo)	6 months	45 patients 2 g/day curcumin + mesalazine or sulfalazine/44 patients’ placebo only drug	Curcumin combinaton with drug-reduced relapses vs placebo Improved CAI, EI
Phosphatidylcholine					
Ehehalt et al. [73]	Rectal mucus of patients with UC and CD (clinical remmision)	CD *n* = 7 UC *n* = 11 Control *n* = 21	–	Mass spectrometric analyses of mucus. Quantitative analyses of phosphatidylcholine (PC) and lysophosphatidylcholine (LPS)	Patients with UC: significant less PC and LPS in mucus compared to CD and control
Treede et al. [75]	CaCo_2_ cells	–	–	Phosphatidylcholine	Inhibition TNF-alfa Reduced: IL-8, ICAM-1, IP-10, MCP-1, TNF-alpha and MMP-1
Amadei et al. [76]	In vitro intestinal surface model	–	–	Phosphatidylcholine	Sustained the binding capability to enzymatically degraded mucin
Stremmel et al. [77]	Patients UC	*n* = 60 (*n* = 30 study group, *n* = 30 placebo group)	3 months	Phosphatidylcholine (PC) supplement 6 g/day vs placebo	Induction of clinical remission in study group CAI index improvement Improvement in quality of life
*Terminalia arjuna*					
Cota et al. [79]	Rat with experimentally induced colitis	*n* = 48 (6 groups *n* = 8 in each group (2 control group: noncolitic, untreated colitic and 4 study group)	28 days	*Terminalia arjuna* (hydroalcoholic extract TAHA) in various doses (500, 250, 125 mg/kg)	Reduction macroscopic and histologic score Decreased MPO, NO, IL-6, IL-1beta, TNF-α, MCP-1
Soy protein					
Metzger et al. [81]	Rats with induced colitis	*n* = 32	28 days	35% soy protein diet	TNF-α reduce Improvement histological examinations in intestinal tissue and bone tissue
Common nettle					
Nematgorgani et al. [84]	Patients with IBD	*n* = 64 (59 ukończyło badanie) (*n* = 30 study group; *n* = 29 placebo group)	3 months	400 mg per tablet nettle extract 3 times/day or placebo group	Reduction CRP serum levels Increase SOD Quality of life improved
Francišković et al. [82]	Ex vivo human platelets	–	–	200 mg/mL nettle extract	Inhibited: thromboxane, LPS production, expression COX-2 Inhibited 12-LOX trail Supports the maintenance of epithelial integrity and intestinal mucosal defensive ability

## Structure of the underlying research

**Fig. 2:**
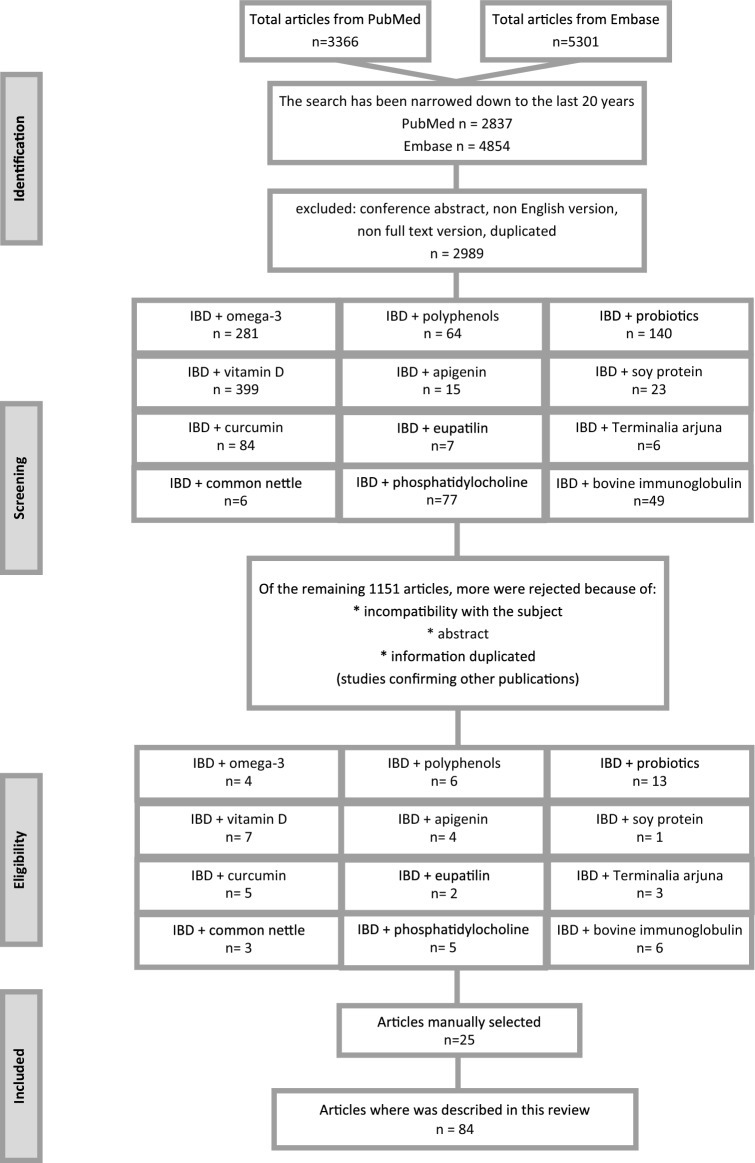
Flow-chart of the review

The present review evaluates the above-mentioned topics considering the literature published up to 31 January 2020. A systematic literature search has been conducted based in the PubMed and Embase database. The passwords were checked on terms: inflammatory bowel diseases, ulcerative colitis and Crohn's disease. These terms were combined with nutrition, supplementation, diet, probiotics ad inflammation. Studies that were not in English language, letters to editor, and abstracts to conferences were excluded as shown on flow chart (Fig. 2). All included studies were screened and discussed by the authors until a general consensus was reached.

## References

[1] Kordjazy N, Haj-Mirzaian A, Haj-Mirzaian A (2018). Role of toll-like receptors in inflammatory bowel disease. Pharmacol Res.

[2] Ashton JJ, Ennis S, Beattie RM (2017). Early-onset paediatric inflammatory bowel disease. Lancet Child Adolesc Health.

[3] Ramos GP, Papadakis KA (2019). Mechanisms of disease: inflammatory bowel diseases. Mayo Clin Proc.

[4] Zhang Y-Z, Li Y-Y (2014). Inflammatory bowel disease: pathogenesis. World J Gastroenterol.

[5] Lewis JD, Abreu MT (2017). Diet as a trigger or therapy for inflammatory bowel diseases. Gastroenterology.

[6] Hooper KM, Barlow PG, Stevens C, Henderson P (2017). Inflammatory bowel disease drugs: a focus on autophagy. J Crohns Colitis.

[7] Radwan P (2008). Leczenie Farmakologiczne choroby Leśniowskiego-Crohna. Choroba Leśniowskiego-Crohna-100 lat diagnostyki i terapii.

[8] Larussa T, Imeneo M, Luzza F (2017). Potential role of nutraceutical compounds in inflammatory bowel disease. World J Gastroenterol.

[9] Julia C, Meunier N, Touvier M (2013). Dietary patterns and risk of elevated C-reactive protein concentrations 12 years later. Br J Nutr.

[10] Sobiś J, Kunert Ł, Sołtysik M (2015). Omega-3 polyunsaturated fatty acids in the prevention of affective disorders. Selected epidemiological data concering the use of omega-3 in the prevention of affective disorders. Psychiatria.

[11] Akbaraly TN, Shipley MJ, Ferrie JE (2015). Long-term adherence to healthy dietary guidelines and chronic inflammation in the prospective Whitehall II study. Am J Med.

[12] Lordan R, Tsoupras A, Zabetakis I (2017). Phospholipids of animal and marine origin: structure, function, and anti-inflammatory properties. Molecules.

[13] Shen C, Dillissen E, Kasran A (2006). Anti-inflammatory activity of a pteridine derivative (4AZA2096) alleviates TNBS-induced colitis in mice. J Interferon Cytokine Res.

[14] Piotrowska A, Iżykowska I, Podhorska-Okołów M (2008). The structure of NF-kB family proteins and their role in apoptosis. Postępy Higieny i Medycyny Doświadczalnej.

[15] Ahmed S, Dewan MZ, Xu R (2014). Nuclear factor-kappaB in inflammatory bowel disease and colorectal cancer. Am J Dig Dis.

[16] Mazur-Bialy AI, Buchala B, Plytycz B (2013). Riboflavin deprivation inhibits macrophage viability and activity—a study on the RAW 264.7 cell line. Br J Nutr.

[17] Szeliga J, Sóndka Z, Jackowski M (2007). The outline of immunopathogenesis of Crohn’s disease with special consideration of NOD2/CARD15 gene polymorphism. Gastroenteroloia Polska.

[18] Head K, Jurenka JS (2004). Inflammatory bowel disease. Part II: Crohn’s disease—pathophysiology and conventional and alternative treatment options. Altern Med Rev.

[19] Guirado A, López Sánchez JI, Ruiz-Alcaraz AJ (2013). First synthesis and biological evaluation of 4-amino-2-aryl-6,9-dichlorobenzo[g]pteridines as inhibitors of TNF-α and IL-6. Eur J Med Chem.

[20] Jenny M, Klieber M, Zaknun D (2011). In vitro testing for anti-inflammatory properties of compounds employing peripheral blood mononuclear cells freshly isolated from healthy donors. Inflamm Res.

[21] Husain N, Tokoro K, Popov JM (2013). Neopterin concentration as an index of disease activity in Crohn’s disease and ulcerative colitis. J Clin Gastroenterol.

[22] Maier E, Kurz K, Jenny M (2010). Food preservatives sodium benzoate and propionic acid and colorant curcumin suppress Th1-type immune response in vitro. Food Chem Toxicol.

[23] Cıralı C, Ulusoy E, Kume T, Arslan N (2018). Elevated serum neopterin levels in children with functional constipation: association with systemic proinflammatory cytokines. World J Pediatr.

[24] Guslandi M, Mezzi G, Sorghi M, Testoni PA (2000). *Saccharomyces boulardii* in maintenance treatment of Crohn’s disease. Dig Dis Sci.

[25] Bourreille A, Cadiot G, Le Dreau G (2013). *Saccharomyces boulardii* does not prevent relapse of Crohn’s disease. Clin Gastroenterol Hepatol.

[26] Sichetti M, De Marco S, Pagiotti R (2018). Anti-inflammatory effect of multistrain probiotic formulation (*L. rhamnosus*, *B. lactis*, and *B. longum*). Nutrition.

[27] Fedorak RN, Feagan BG, Hotte N (2015). The probiotic VSL#3 has anti-inflammatory effects and could reduce endoscopic recurrence after surgery for Crohn’s disease. Clin Gastroenterol Hepatol.

[28] Le B, Yang SH (2018). Efficacy of *Lactobacillus plantarum* in prevention of inflammatory bowel disease. Toxicol Rep.

[29] Pathmakanthan S, Li CKF, Cowie J, Hawkey CJ (2004). *Lactobacillus plantarum* 299: beneficial in vitro immunomodulation in cells extracted from inflamed human colon. J Gastroenterol Hepatol.

[30] Wu S, Yoon S, Zhang Y-G (2015). Vitamin D receptor pathway is required for probiotic protection in colitis. Am J Physiol Gastrointest Liver Physiol.

[31] Ahn Y-S, Park MY, Shin J-H (2014). Lysate of probiotic *Lactobacillus plantarum* K8 modulate the mucosal inflammatory system in dextran sulfate sodium-induced colitic rats. Korean J Food Sci Anim Resour.

[32] Yin M, Yan X, Weng W (2018). Micro integral membrane protein (MIMP), a Newly discovered anti-inflammatory protein of *Lactobacillus plantarum*, enhances the gut barrier and modulates microbiota and inflammatory cytokines. Cell Physiol Biochem.

[33] Levit R, Savoy de Giori G, de Moreno de LeBlanc A, LeBlanc JG, (2018). Protective effect of the riboflavin-overproducing strain *Lactobacillus plantarum* CRL2130 on intestinal mucositis in mice. Nutrition.

[34] Derwa Y, Gracie DJ, Hamlin PJ, Ford AC (2017). Systematic review with meta-analysis: the efficacy of probiotics in inflammatory bowel disease. Aliment Pharmacol Ther.

[35] Kruis W, Frič P, Pokrotnieks J (2004). Maintaining remission of ulcerative colitis with the probiotic *Escherichia coli* Nissle 1917 is as effective as with standard mesalazine. Gut.

[36] Probiotics and Prebiotics|World Gastroenterology Organisation. https://www.worldgastroenterology.org/guidelines/global-guidelines/probiotics-and-prebiotics. Accessed 9 Dec 2019

[37] Shafran I, Burgunder P, Wei D (2015). Management of inflammatory bowel disease with oral serum-derived bovine immunoglobulin. Therap Adv Gastroenterol.

[38] Beauerle BD, Burnett BP, Dryden GW (2015). Successful management of refractory ulcerative colitis with orally administered serum-derived bovine immunoglobulin therapy. Clin Case Rep Rev.

[39] Shaw AL, Tomanelli A, Bradshaw TP (2017). Impact of serum-derived bovine immunoglobulin/protein isolate therapy on irritable bowel syndrome and inflammatory bowel disease: a survey of patient perspective. Patient Prefer Adher.

[40] Liaquat H, Ashat M, Stocker A (2018). Clinical efficacy of serum-derived bovine immunoglobulin in patients with refractory inflammatory bowel disease. Am J Med Sci.

[41] Soriano RA, Ramos-Soriano AG (2017). Clinical and pathologic remission of pediatric ulcerative colitis with serum-derived bovine immunoglobulin added to the standard treatment regimen. CRG.

[42] Shafran I, Young HE, Wei D (2016). Pilot pharmacoeconomic analysis of serum-derived bovine immunoglobulin use in IBD. Am J Pharm Benefits.

[43] Alhassan Mohammed H, Mirshafiey A, Vahedi H (2017). Immunoregulation of inflammatory and inhibitory cytokines by vitamin D3 in patients with inflammatory bowel diseases. Scand J Immunol.

[44] Del Pinto R, Ferri C, Cominelli F (2017). Vitamin D axis in inflammatory bowel diseases: role, current uses and future perspectives. Int J Mol Sci.

[45] Lin Z, Li W (2016). The roles of vitamin D and its analogs in inflammatory diseases. Curr Top Med Chem.

[46] Blanck S, Aberra F (2013). Vitamin D deficiency is associated with ulcerative colitis disease activity. Dig Dis Sci.

[47] Laird E, McNulty H, Ward M (2014). Vitamin D deficiency is associated with inflammation in older Irish adults. J Clin Endocrinol Metab.

[48] Jørgensen SP, Agnholt J, Glerup H (2010). Clinical trial: vitamin D3 treatment in Crohn’s disease—a randomized double-blind placebo-controlled study. Aliment Pharmacol Ther.

[49] Del Pinto R, Pietropaoli D, Chandar AK (2015). Association between inflammatory bowel disease and vitamin D deficiency: a systematic review and meta-analysis. Inflamm Bowel Dis.

[50] Forbes A, Escher J, Hébuterne X (2017). ESPEN guideline: clinical nutrition in inflammatory bowel disease. Clin Nutr.

[51] He K, Liu K, Daviglus ML (2009). Associations of dietary long-chain n-3 polyunsaturated fatty acids and fish with biomarkers of inflammation and endothelial activation (from the Multi-Ethnic Study of Atherosclerosis [MESA]). Am J Cardiol.

[52] Charpentier C, Chan R, Salameh E (2018). Dietary n-3 PUFA may attenuate experimental colitis. Mediators Inflamm.

[53] Mbodji K, Charpentier C, Guérin C (2013). Adjunct therapy of n-3 fatty acids to 5-ASA ameliorates inflammatory score and decreases NF-κB in rats with TNBS-induced colitis. J Nutr Biochem.

[54] Turner D, Shah PS, Steinhart AH (2011). Maintenance of remission in inflammatory bowel disease using omega-3 fatty acids (fish oil): a systematic review and meta-analyses. Inflamm Bowel Dis.

[55] Oledzka R (2007). Nutraceutyki, zywnosc funkcjonalna—rola i bezpieczenstwo stosowania. Bromatologia i Chemia Toksykologiczna.

[56] Joo M, Kim HS, Kwon TH (2015). Anti-inflammatory effects of flavonoids on TNBS-induced colitis of rats. Korean J Physiol Pharmacol.

[57] Zhou K, Cheng R, Liu B (2018). Eupatilin ameliorates dextran sulphate sodium-induced colitis in mice partly through promoting AMPK activation. Phytomedicine.

[58] Włochal M, Grzymisławski M (2016). New trends in the dietary treatment of inflammatory bowel diseases. Pielęgniarstwo i Zdrowie Publiczne Nurs Public Health.

[59] Mascaraque C, González R, Suárez MD (2015). Intestinal anti-inflammatory activity of apigenin K in two rat colitis models induced by trinitrobenzenesulfonic acid and dextran sulphate sodium. Br J Nutr.

[60] Sadraei H, Asghari G, Khanabadi M, Minaiyan M (2017). Anti-inflammatory effect of apigenin and hydroalcoholic extract of *Dracocephalum kotschyi* on acetic acid-induced colitis in rats. Res Pharm Sci.

[61] Ben-Arye E, Goldin E, Wengrower D (2002). Wheat grass juice in the treatment of active distal ulcerative colitis: a randomized double-blind placebo-controlled trial. Scand J Gastroenterol.

[62] Boussenna A, Cholet J, Goncalves-Mendes N (2016). Polyphenol-rich grape pomace extracts protect against dextran sulfate sodium-induced colitis in rats. J Sci Food Agric.

[63] D’Argenio G, Mazzone G, Tuccillo C (2012). Apple polyphenols extract (APE) improves colon damage in a rat model of colitis. Dig Liver Dis.

[64] Parkar SG, Stevenson DE, Skinner MA (2008). The potential influence of fruit polyphenols on colonic microflora and human gut health. Int J Food Microbiol.

[65] Sun X, Du M, Navarre DA, Zhu M-J (2018). Purple potato extract promotes intestinal epithelial differentiation and barrier function by activating AMP-activated protein kinase. Mol Nutr Food Res.

[66] Muanprasat C, Sirianant L, Sawasvirojwong S (2013). Activation of AMP-activated protein kinase by a plant-derived dihydroisosteviol in human intestinal epithelial cell. Biol Pharm Bull.

[67] Sun X, Yang Q, Rogers CJ (2017). AMPK improves gut epithelial differentiation and barrier function via regulating Cdx2 expression. Cell Death Differ.

[68] Suskind DL, Wahbeh G, Burpee T (2013). Tolerability of curcumin in pediatric inflammatory bowel disease: a forced-dose titration study. J Pediatr Gastroenterol Nutr.

[69] Garg SK, Ahuja V, Sankar MJ (2012). Curcumin for maintenance of remission in ulcerative colitis. Cochrane Database Syst Rev.

[70] Iqbal U, Anwar H, Quadri AA (2018). Use of curcumin in achieving clinical and endoscopic remission in ulcerative colitis: a systematic review and meta-analysis. Am J Med Sci.

[71] Hanai H, Iida T, Takeuchi K (2006). Curcumin maintenance therapy for ulcerative colitis: randomized, multicenter, double-blind, placebo-controlled trial. Clin Gastroenterol Hepatol.

[72] Jiménez-Flores LM, López-Briones S, Macías-Cervantes MH (2014). A PPARγ, NF-κB and AMPK-dependent mechanism may be involved in the beneficial effects of curcumin in the diabetic db/db mice liver. Molecules.

[73] Ehehalt R, Wagenblast J, Erben G (2004). Phosphatidylcholine and lysophosphatidylcholine in intestinal mucus of ulcerative colitis patients. A quantitative approach by nanoElectrospray-tandem mass spectrometry. Scand J Gastroenterol.

[74] Vetter M, Neurath MF (2017). Emerging oral targeted therapies in inflammatory bowel diseases: opportunities and challenges. Therap Adv Gastroenterol.

[75] Treede I, Braun A, Jeliaskova P (2009). TNF-alpha-induced up-regulation of pro-inflammatory cytokines is reduced by phosphatidylcholine in intestinal epithelial cells. BMC Gastroenterol.

[76] Amadei F, Fröhlich B, Stremmel W, Tanaka M (2018). Nonclassical interactions of phosphatidylcholine with mucin protect intestinal surfaces: a microinterferometry study. Langmuir.

[77] Stremmel W, Merle U, Zahn A (2005). Retarded release phosphatidylcholine benefits patients with chronic active ulcerative colitis. Gut.

[78] Amalraj A, Gopi S (2017). Medicinal properties of *Terminalia arjuna* (Roxb.) Wight & Arn.: a review. J Tradit Complement Med.

[79] Cota D, Mishra S, Shengule S (2019). Beneficial role of *Terminalia arjuna* hydro-alcoholic extract in colitis and its possible mechanism. J Ethnopharmacol.

[80] Ahmad MS, Ahmad S, Gautam B (2014). *Terminalia arjuna*, a herbal remedy against environmental carcinogenicity: an in vitro and in vivo study. Egypt J Med Hum Genet.

[81] Metzger CE, Narayanan SA, Zawieja DC, Bloomfield SA (2019). A moderately elevated soy protein diet mitigates inflammatory changes in gut and in bone turnover during chronic TNBS-induced inflammatory bowel disease. Appl Physiol Nutr Metab.

[82] Francišković M, Gonzalez-Pérez R, Orčić D (2017). Chemical composition and immuno-modulatory effects of *Urtica dioica* L. (Stinging Nettle) extracts. Phytother Res.

[83] Emsley J (2011). Nature’s building blocks: an A–Z guide to the elements.

[84] Nematgorgani S, Agah S, Shidfar F (2017). Effects of *Urtica dioica* leaf extract on inflammation, oxidative stress, ESR, blood cell count and quality of life in patients with inflammatory bowel disease. J Herb Med.

